# Watchful waiting versus pharmacological management of small-for-gestational-age infants with hyperinsulinemic hypoglycemia

**DOI:** 10.3389/fendo.2023.1163591

**Published:** 2023-06-14

**Authors:** Suresh Chandran, Sandra Lynn Jaya-Bodestyne, Victor Samuel Rajadurai, Seyed Ehsan Saffari, Mei Chien Chua, Fabian Yap

**Affiliations:** ^1^ Department of Neonatology, Kandang Kerbau (KK) Women’s and Children’s Hospital, Singapore, Singapore; ^2^ Pediatric Academic Clinical Programme, Lee Kong Chian School of Medicine, Singapore, Singapore; ^3^ Pediatric Academic Clinical Programme, Duke-NUS Medical School, Singapore, Singapore; ^4^ Pediatric Academic Clinical Programme, Yong Loo Lin School of Medicine, Singapore, Singapore; ^5^ Center for Quantitative Medicine, Office of Clinical Science, Duke-NUS Medical School, Singapore, Singapore; ^6^ Department of Pediatric Endocrinology, Kandang Kerbau (KK) Women’s and Children’s Hospital, Singapore, Singapore

**Keywords:** diazoxide, hyperinsulinemic hypoglycemia, fasting study, small-for-gestational-age, watchful waiting

## Abstract

**Introduction:**

Given that reports on severe diazoxide (DZX) toxicity are increasing, we aimed to understand if the short-term clinical outcomes of small-for-gestational-age (SGA) infants with hyperinsulinemic hypoglycemia (HH) managed primarily by supportive care, termed watchful waiting (WW), are different from those treated with DZX.

**Method:**

A real-life observational cohort study was conducted from 1 September 2014 to 30 September 2020. The WW or DZX management decision was based on clinical and biochemical criteria. We compared central line duration (CLD), postnatal length of stay (LOS), and total intervention days (TIDs) among SGA-HH infants treated with DZX versus those on a WW approach. Fasting studies determined the resolution of HH.

**Result:**

Among 71,836 live births, 11,493 were SGA, and 51 SGA infants had HH. There were 26 and 25 SGA-HH infants in the DZX and WW groups, respectively. Clinical and biochemical parameters were similar between groups. The median day of DZX initiation was day 10 of life (range 4–32), at a median dose of 4 mg/kg/day (range 3–10). All infants underwent fasting studies. Median CLD [DZX, 15 days (6–27) vs. WW, 14 days (5–31), P = 0.582] and postnatal LOS [DZX, 23 days (11–49) vs. WW, 22 days (8–61), P = 0.915] were comparable. Median TID was >3-fold longer in the DZX than the WW group [62.5 days (9–198) vs. 16 days (6–27), P < 0.001].

**Conclusion:**

CLD and LOS are comparable between WW and DZX groups. Since fasting studies determine the resolution of HH, physicians should be aware that clinical intervention of DZX-treated SGA-HH patients extends beyond the initial LOS.

## Introduction

Many small-for-gestational-age (SGA) infants are substrate deficient and, therefore, at risk of hypoglycemia (1). Since the likelihood of developing hypoglycemia is greatest in the first hours of life when the physiological glucose nadir occurs, SGA babies need to be fed soon after birth and monitored (2). Other mechanisms of hypoglycemia include hyperinsulinemic hypoglycemia (HH), impaired gluconeogenesis/glycogenolysis, and adrenocortical insufficiency (3, 4). Approximately 1 in 200 SGA infants will develop HH during neonatal life, primarily attributed to perinatal stress hyperinsulinism (PSHI), which is usually transient (5, 6). Yet, at initial presentation, it is clinically difficult to predict if the temporal nature of HH will be transient, prolonged, or persistent and whether the etiology will be genetic or not.

Clinicians managing SGA-HH infants are challenged in two ways. The first question is whether HH will be prolonged or persistent since PSHI has been reported to continue from 18 to 403 days or if it will be transient and spontaneously resolve within a week (7, 8). The second challenge relates to pharmacotherapy, as it is difficult to predict the SGA-HH infant who will benefit from diazoxide (DZX) therapy while at the same time identifying those at the highest risk of developing side effects (5). Clinicians who favor pharmacotherapy are often persuaded by the economic consequences of prolonged LOS and the fear of litigation (9). On the other hand, the growing list of published side effects of DZX therapy warrants caution (10, 11). Furthermore, whether SGA-HH babies treated with DZX fare better than those managed more conservatively without pharmacotherapy remains fundamentally unclear.

We hypothesized that the short-term outcomes of SGA-HH infants treated with DZX are not better than those managed by watchful waiting (WW). The primary aim of our study was to compare the central venous line duration (CLD), length of stay (LOS), and total intervention days (TIDs) till resolution of HH among watchful waiting (WW group) versus DZX-treated (DZX group) SGA infants with HH.

## Materials and methods

### Study setting and patient population

**Figure 1:**
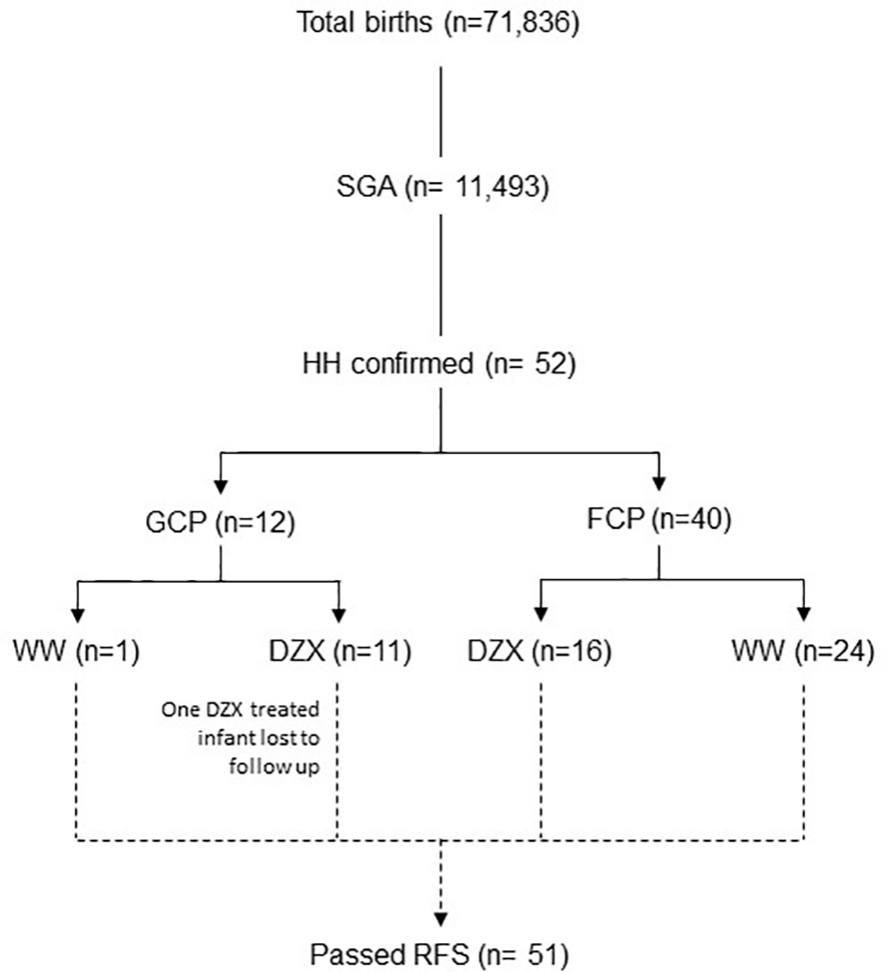
The flow diagram of participants included in the study. DZX, diazoxide; HH, hyperinsulinemic hypoglycemia; FCP; feed-centric pathway; GCP, glucose-centric pathway; RFS, resolution fasting study; SGA, small-for-gestational-age; WW, watchful waiting.

This observational cohort study was conducted in a real-life setting over 73 months from 1 September 2014 to 30 September 2020 at KK Women’s and Children’s Hospital Singapore, a national tertiary referral center with an annual birth rate of approximately 11,000 per year. All SGA infants with HH during the study period were included. We included infants in the intensive care ward who were admitted primarily for central line HH management and had no other major medical or surgical conditions. We excluded infants who did not have a fasting study that demonstrated resolution (**Figure 1**). This study was granted exemption from ethics approval by the Centralized Institutional Review Board (CIRB 2021/2166).

### Definitions

We defined SGA as those with a birth weight of <10th centile for gestational age using the Fenton reference (12). We defined hypoglycemia according to the pathway in use (glucose-centric or feed-centric pathway), as detailed below. We confirmed HH in any infant >48 h of life with (a) hypoglycemia (<3.0 mmol/L), (b) inappropriate insulin levels (≥ 1.6 mU/L (chemiluminescence microparticle immunoassay, Abbott, USA), (c) hypoketonemia (<0.6 mmol/L), and (d) hypofattyacidemia (<0.5 mmol/L) (13). We defined the resolution of HH by fasting studies (14, 15).

### Hypoglycemia screening

Glucose screening was performed using a bedside glucometer (Abbott Optium Neo, UK). Laboratory determination of plasma glucose is performed when the glucometer reading is <3 mmol/L. All critical blood samples had paired plasma glucose with serum insulin.

Two hypoglycemia screening methods were used during the study period. The first method was a glucose-centric pathway (GCP), where blood glucose soon after birth directed the subsequent management actions. In a GCP, hypoglycemia was defined as ≤2.5 mmol/L. The GCP period covered 17 months, from 1 September 2014 to 31 January 2016. The second method was a feed-centric pathway (FCP), which instead focused on early feeding, skin-to-skin care, and glucose testing at 2 h of life. In an FCP, hypoglycemia was defined as <3 mmol/L. The FCP period covered the remaining 56 months of the study period from 1 February 2016 to 30 September 2020. The key features of the GCP and FCP are presented in **Table 1**, details of which were previously published (16).

**Table 1:** Common and unique features of the glucose-centric pathway (GCP) and feed-centric pathway (FCP).

	GCP	FCP
Common features	1. Provide feeds in the first 48 h of life 2. Monitor blood glucose in the first 24 h of life in all at-risk infants 3. Provide IV dextrose for symptomatic infants 4. Critical sampling after 48 h of life 5. Critical blood sampling threshold is GIR >10mg/kg/min	
Unique features		
Target blood glucose (mmol/L)	≤2.5	<3
Time of glucose testing (hour of life)	0, 1, 3, 6, 12, 24	2, 6, 12, 18, 24, 36
Approach to intervention for asymptomatic infants	Step 1: IV dextrose/feeds Step 2: IV glucagon Step 3: IV hydrocortisone Step 4: Consider diazoxide	Step 1: Skin-to-skin and breastfeeding Step 2: Donor or formula milk Step 3: Buccal glucose gel Step 4: IV dextrose Step 5: Consider diazoxide

### Hypoglycemia management

The approach to hypoglycemia management was pathway-specific, as described in **Table 1**. The principal feature in common between the GCP and FCP was the focus on providing substrate in the first 48 h and testing for HH only after 48 h in those with the glucose infusion rate (GIR) >10 mg/kg/min. The maximum GIR was recorded in our cohort before DZX initiation. In contrast to the GCP, the unique feature of the FCP was the starting point of care that included a universal skin-to-skin approach, breastfeeding first, followed by buccal glucose [40% glucose gel; dose 200 mg/kg (0.5 ml/kg)] up to two doses given 30 min apart) when required, and restricting the use of intravenous (IV) dextrose as far as possible (16). In the FCP, the use of IV glucagon was avoided, and IV hydrocortisone was used only when adrenal insufficiency was proven.

### Diazoxide

The decision to commence DZX was based on clinical and biochemical criteria throughout the study. The criteria to consider DZX therapy was previously published (5).

(a) Recurrent need to increase GIR to maintain normoglycemia after 48 h of age.
(b) Recurrent episodes of low blood glucose despite increasing feeds while weaning IV glucose.
(c) Inability to achieve full feeds by 1–2 weeks of age while attempting to reduce IV glucose.

Normal full blood counts, liver and renal function parameters, and absence of pericardial effusion and pulmonary hypertension in an echocardiogram were prerequisites to start DZX. We started DZX at a dose of 3 mg/kg/day in two divided doses and increased at 2–2.5 mg/kg/day if the initial dose response was inadequate. As we have previously published, a lower starting dose was chosen (compared to the conventional dose of 5–20 mg/kg/day) based on its efficacy in treating HH in SGA infants with minimal adverse effects (5). All infants were provided with oral hydrochlorothiazide at a dose of 1 mg/kg/day in two divided doses to mitigate the fluid-retaining side effect of DZX. Once adequate response to DZX was obtained, as defined by normal pre-feed glucose for 48 h on full oral feeds, we performed a 6-h safety fasting study (SFS) before discharging home. An infant is considered to pass the fasting study if glucose levels are normal during the fasting period and at the end of the study. The SFS was designed to confirm the infant’s ability to maintain glucose levels during an inadvertent fast at home. Home glucose monitoring was continued until the dose of DZX was passively weaned (with weight gain) to half the initial dose or <1.5 mg/kg/day. An active weaning of DZX was done if glucose levels were above >7 mmol/L. Infants on home glucose monitoring were tasked to inform the nurse of any hypoglycemia episodes. Subsequently, to allow clearance of DZX from the circulation, medications were discontinued for 3 days while closely monitoring symptoms and glucose levels at home. On day 4 of DZX discontinuation, the infant was admitted to the hospital for a supervised age-appropriate resolution fasting study (RFS) (5). Passing RFS qualified the infant to remain off DZX and continue with growth and neurodevelopmental follow-up. None of the DZX-treated participants required readmission for hypoglycemia after RFS.

### Watchful waiting

Infants who did not receive DZX therapy were on a WW approach. We performed a 6-h fasting study before discharge once WW infants were taken off parenteral glucose and had stable pre-feed glucose for 48 h while on full oral feeds. Passing this fasting study qualified the infant safe for discharge home with parental education, feeding training, observation for hypoglycemia symptoms, and safe use of buccal glucose. This fasting study was considered RFS for WW infants and defined the resolution of HH and the presence of an intact hormone regulation mechanism to maintain normoglycemia. None of the WW participants required readmission for hypoglycemia after RFS.

### Outcome measures

Data collected on infants included gender, gestational age at birth, birth weight, age at presentation, symptoms, critical investigations (plasma glucose and paired glucose and insulin levels), and highest GIR. Information related to DZX included the day of initiation, the highest dose administered, and the duration of treatment. CLD, LOS, and TID were recorded as outcome measures. CLD was the interval between the insertion of the umbilical or peripherally inserted central line and its removal. LOS was the duration from birth to the day of discharge. TID was the time interval from the day of diagnosis of HH to the day when the patient passed the RFS.

Statistical analysis

We performed statistical analysis in SAS software version 9.4 for Windows (Cary, NC: SAS Institute Inc.). Demographics and clinical features were reported as descriptive statistics via frequency and percentage for categorical variables and mean ± standard deviation and median (minimum–maximum) for continuous variables. The two groups (DZX and WW) were compared using Fisher’s exact test and the Mann–Whitney U test for categorical and continuous variables. The Mann–Whitney U test was also conducted to compare DZX versus WW. Due to small sample sizes per group, the normality assumption may not be tenable and non-parametric methods are used. Exploratory analysis was performed to adjust for the time of diagnosis (GCP and FCP) using a multivariable generalized linear model for the clinical outcomes, and the adjusted p values were calculated. Statistical significance was set at p < 0.05.

## Results

**Figure 2:**
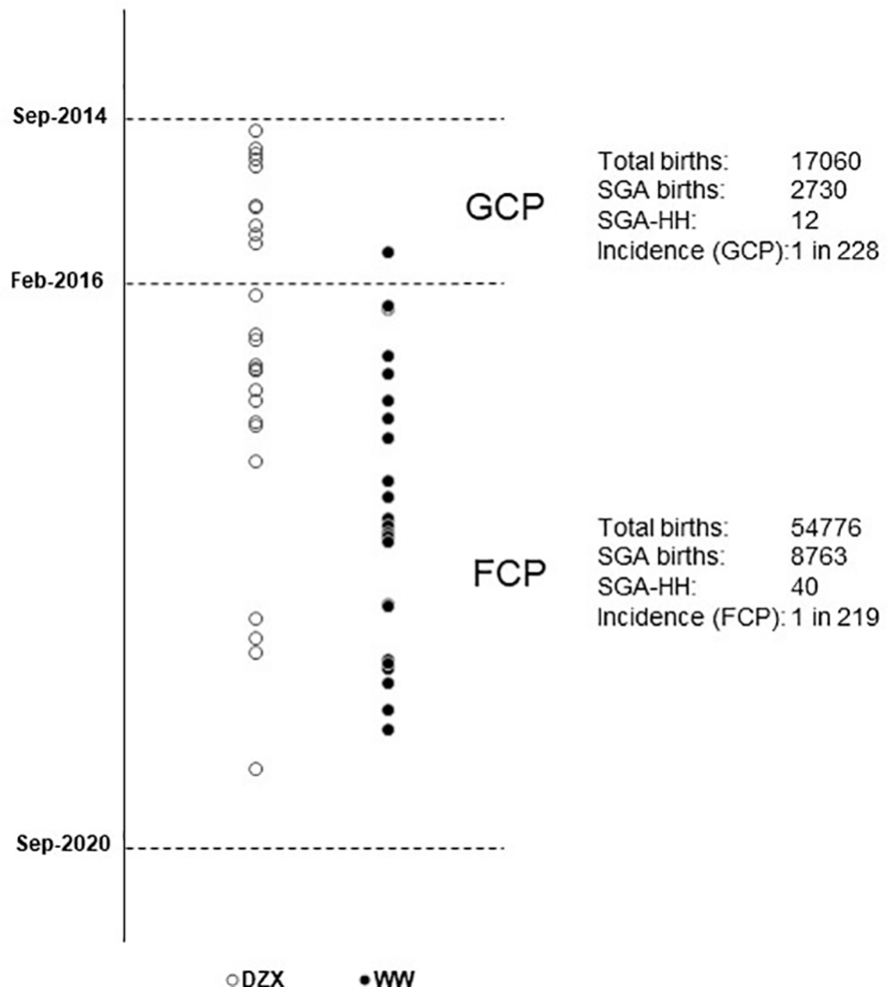
Vertical timeline of SGA infants diagnosed with HH during the study period, managed with DZX (n = 26, open ο circles) and WW (n = 25, bold • circles). The respective incidence of SGA-HH during the glucose-centric pathway (GCP) and the feed-centric pathway (FCP) period is shown. DZX, diazoxide; HH, hyperinsulinemic hypoglycemia; SGA, small-for-gestational-age; WW, watchful waiting.

**Figure 2** depicts the timeline when SGA infants were consecutively diagnosed with HH during the study period and categorized by whether they received DZX or were managed by WW. Excluding one infant in the DZX group who returned overseas, there were 51 SGA infants with HH among 11,493 with SGA from 71,836 live births, giving an overall SGA-HH incidence of 1 in 225. The incidence of SGA-HH was similar in the GCP (1 in 228) and FCP (1 in 219) groups.

**Table 2** describes the clinical characteristics of the 51 SGA infants with HH, of which 26 received DZX treatment and 25 had WW. Sex, gestational age, birth weight, day of presentation, plasma glucose level at presentation, day of diagnosis of HH, and paired glucose/insulin level were not different between DZX and WW infants. In the DZX group, more infants had symptoms, and their maximum GIR was higher. However, these were not statistically significant. All infants in the DZX group received DZX after confirmation of HH.

**Table 2:** Clinical characteristics of hyperinsulinemic hypoglycemia infants treated with DZX versus WW.

Characteristic	DZX group (n = 26)	WW group (n = 25)	P value^*^
Sex (male)	15 (60%) ^†^	14 (53.9%) ^†^	0.779
Gestational age (weeks)	36.5 ± 2.1 37 (31–40)	36.1 ± 2.4 36 (30–39)	0.688
Birthweight (g)	1,948 ± 362 2,025 (1,120–2,370)	1,932 ± 500 1,915 (900–2,655)	0.985
Symptom(s) at presentation	Jittery 5 (19.2%) Seizure 1 (3.9%)	Jittery 2 (8%) Seizure 0 (0%)	0.330
Day of presentation	1.1 ± 0.4 1 (1–3)	1.0 ± 0.2 1.0 (1–2)	1.000
Day of diagnosis of HH	6.6 ± 3.3 6 (3–18)	6.2 ± 2.0 6 (3–12)	0.954
Plasma glucose (mmol/L)	1.3 ± 0.7 1.4 (0.3–2.9)	1.3 ± 0.6 1.2 (0.3–2.6)	0.828
Paired glucose/insulin levels (mmol/L)/(mU/L) ^#^	2.4 ± 0.4/11.7 ± 12.8 2.6 (1.4–2.9)/4.6 (1.9–53)	2.2 ± 0.4/9 ± 11.2 2.3 (1.2–2.9)/6.9 (1.6–57.6)	0.051/0.734
Maximum GIR (mg/kg/min)	15.2 ± 4.2 14.4 (10–26.2)	13.7 ± 2.8 13 (10–20.3)	0.187

†Numeric variables are reported as mean ± standard deviation and median (minimum–maximum); categorical variables are reported as frequency (percent).

*The DZX and WW groups were compared using Fisher’s exact test and the Mann–Whitney U test for categorical and continuous variables, respectively.

#Paired glucose/insulin levels measured after 48 h of life as part of critical samples to diagnose hyperinsulinism DZX, diazoxide; GIR, glucose infusion rate; HH, hyperinsulinemic hypoglycemia; WW, watchful waiting.

The median dose (mg/kg/day) of DZX received by infants in the DZX group was 4 (range 3–10). All infants in the DZX group underwent SFS, and all had RFS. In the WW group, all had documentation of the resolution of HH by RFS.

**Table 3** demonstrates the clinical outcomes. The median day of DZX initiation was day 10 of life (range 4–32). Median CLD (DZX 15 days, range 6–27 vs. WW 14 days, range 5–31, P = 0.582) and postnatal LOS (DZX 23 days, range 11–49 vs. WW 22 days, range 8–61, P = 0.915) were statistically comparable between the groups. However, the median TID was 16 days (range 6–27) in the WW group, in contrast to 62.5 days (range 9–198) (P < 0.001) in the DZX group (**Table 3**). The results of the exploratory multivariable model adjusting for the time of diagnosis are comparable with the above findings (CLD-adjusted P value = 0.268; LOS adjusted P value = 0.972; TID-adjusted P value < 0.001).

**Table 3:** Clinical outcomes of infants treated with DZX versus WW.

Variables	DZX group (n = 26)	WW group (n = 25)	P value^*^	Adjusted P value^**^
Central line duration (days)	15.5 ± 5.6 15 (6–27)	14.8 ± 6.7 14 (5–31)	0.582	0.268
Length of stay (days)	23.2 ± 9.7 23 (11–49)	24.3 ± 13 22 (8–61)	0.915	0.972
Diazoxide initiation (day of life)	13.3 ± 8.1 10 (4–32)	N.A.	N.A.	N.A.
Dose of DZX (mg/kg/day)	4.6 ± 2.1 4 (3–10)	N.A.	N.A.	N.A.
Total intervention days (days)	68 ± 40.6 62.5 (9–198)	16.6 ± 5.9 16 (6–27)	<0.001	<0.001

†Figures reported as mean ± standard deviation and median (minimum – maximum).

*Mann–Whitney U test.

**Multivariable generalized linear model adjusting for time of diagnosis.

DZX, diazoxide; WW, watchful waiting.

Two infants (7.4%) treated with DZX 10 mg/kg/day developed marked generalized hypertrichosis, and another infant (3.7%) on DZX 4.8 mg/kg/day developed hyponatremia with pleural effusion.

For a comparison of the clinical outcomes between infants in the GCP (n = 11) and FCP (n = 40), please refer to **Supplementary Table 1**.

## Discussion

In support of our hypothesis, our results demonstrate that the inclusion of DZX in the management of SGA infants with HH did not significantly reduce nor extend the duration of central venous line placement nor shorten the postnatal LOS, compared to WW. Instead, when defined by fasting studies, DZX use substantially increased the TID by threefold among treated infants compared to those who did not receive the pharmacological intervention. This is because the methodology of DZX cessation typically involves gradual dose reduction with weight gain before cessation (5, 15). We also showed that among non-DZX treated SGA infants treated by WW in our cohort, HH resolved by a median of 16 days and not later than 27 days. We are unaware of a study that demonstrates the duration of SGA-HH in non-pharmacologically treated infants.

Our findings provide pragmatic evidence to medical practitioners that a supportive WW approach does not prolong central venous line requirement nor lengthen the duration of hospital stay compared to DZX- treated infants, also implying that DZX treatment does not shorten these parameters, which contrasts with the findings and conclusions of Balachandran et al. (17). These findings also provide clinicians, who are often faced with clinical pressure to initiate DZX therapy, with real-life clinical data that an alternative management modality is available that spares patients from the potential risks of DZX therapy including necrotizing enterocolitis (10, 11, 18), pericardial effusion (19), and pulmonary hypertension (20, 21). Despite several reports of NEC in DZX-exposed preterm and SGA infants, Gray et al. suggest that it is an association rather than causation (22). The biological mechanism of these side effects may be due to the persistent hyperpolarization of neuronal and intestinal cells since DZX is a K_ATP_ channel agonist (10, 23).

It is established that SGA infants are at risk of impaired cognitive and neurological outcomes, whether they develop hypoglycemia or not (24, 25). Therefore, it is understandable that physicians have litigation concerns (9) and face pressure to provide active treatment when SGA infants develop low blood glucose since it may worsen outcomes and lead to neuronal injuries as a result of potential extensive white matter changes in the brain (26). Yet, current evidence suggests that although hypoglycemia exposure may impair executive function and visual–motor function in early childhood (27), it does not necessarily lead to lower educational achievement in mid-childhood (28). However, Sigal et al. reported that PSHI infants are at high risk of neurodevelopmental deficits and are more likely to perform below average (29).

We also show that the TID was substantially longer when clinicians intervened with DZX because treatment can only safely end when a formal fasting study is performed to document the resolution of the hyperinsulinemic state (15, 30). We appreciate that there may be concerns that our tapering method may lead to prolonged intervention. However, one published report recommends attempting withdrawal of DZX when the dosage is below 1 mg/kg/day. This study by Yorifuji et al. and ours considered safety a priority and an endpoint proven by a fasting study (15). Until an alternative method that satisfies these criteria is described, the tapering method may need to be necessarily adequate. The enthusiasm to initiate DZX is not often balanced with the necessary processes to stop it. Clinicians justify starting DZX by either citing the need to remove central lines to minimize the risk of sepsis or facilitating discharge to reduce the cost of inpatient care. However, our data suggest otherwise. Additionally, we appreciate that the responsiveness to DZX can reassure clinicians of the likely non-genetic nature of HH in SGA infants, as previously demonstrated by Arya et al. (31). Physicians often take calculated risks by stopping DZX without fasting studies to minimize the cost of care (30, 32). Whether this is appropriate is still being determined, as there needs to be a good clinical consensus in the literature on whether it is necessary to prove a resolution and how and when to stop DZX. Furthermore, there are differential costs of ready-made DZX preparations, which are highly expensive, versus compounded liquid formulations, which are less costly and less reliable but require pharmacy expertise (32, 33). We suggest that DZX therapy for SGA-HH should be regarded as a continuum of initiating DZX appropriately, monitoring glucose regularly, and stopping DZX safely (5). Clinical teams need to understand that costs extend beyond the initial LOS and that prolonging the duration of care may paradoxically add to psychological and economic burdens (34).

### Strengths and limitations

To the best of our knowledge, this is the first study to compare clinically relevant outcomes of WW over pharmacological intervention in SGA-HH infants in a demographically and biochemically comparable cohort. We also determined the total duration of clinical commitment the patient and provider needed from diagnosis to HH resolution as determined by fasting studies. We acknowledge that two different pathways were employed during the study period, which limits consistency. However, because care pathways are so variable, presenting data from both pathways has provided practical, real-life information to clinical practitioners. To enhance the generalizability of our findings, we defined SGA by the Fenton 2013 criteria, where we identified 16% of live births as SGA even though we expected 10%. This is because birth norms from a Singapore cohort showed that the 10th percentile superimposed well with the Fenton chart between 25 and 39 weeks of gestation and then separated from 39 to 42 weeks, resulting in overestimation of SGA birth size in these latter babies (35). This limitation would apply to all populations, including Singapore. We did not perform genetic studies as all infants were either DZX responsive or underwent spontaneous resolution. We did not study the economic implications of these treatment modalities apart from establishing the duration of care. We acknowledge that the choice of treatment varied with time, with more recent cases being managed with WW. However, adjustment for time showed comparable findings. Due to the recent nature of this study, longitudinal follow-up is still in progress, and this information would be useful to determine the long-term outcomes.

## Conclusion

This real-world observational study demonstrates that CLD and LOS are comparable among SGA-HH infants, whether DZX treatment is employed or not. Watchful waiting while supporting the metabolic needs of SGA infants with hyperinsulinism is a justifiable alternative to DZX therapy in infants with HH. Since fasting studies determine the resolution of HH, physicians should be aware that clinical intervention of DZX-treated SGA-HH infants extends beyond the initial LOS. However, as this was an observational study and SGA-HH is uncommon, our findings ought to be confirmed in a pragmatic multicenter randomized trial.

## Data availability statement

The original contributions presented in the study are included in the article/**Supplementary Material**. Further inquiries can be directed to the corresponding authors.

## Ethics statement

The studies involving human participants were reviewed and approved by Singhealth Centralised Institutional Review Board, Singapore. Written informed consent from the participants’ legal guardian/next of kin was not required to participate in this study in accordance with the national legislation and the institutional requirements.

## Author contributions

SC and FY conceived and designed the study, interpreted the data, and wrote the final manuscript; SJ-B prepared the preliminary draft of the manuscript, including references; VR designed the study and interpreted the data; SS conducted the statistical analysis and interpreted the data; MC designed the study and interpreted the data. SC and FY contributed equally. All authors critically revised the manuscript, agreed to be fully accountable for ensuring the integrity and accuracy of the work. All authors contributed to the article and approved the submitted version.

## Supplementary material

Supplementary Table 1 Clinical outcomes of infants comparing GCP vs FCP.
