# Supplementary material for: Watchful waiting versus pharmacological management of small-for-gestational-age infants with hyperinsulinemic hypoglycemia
Source: Front Endocrinol (Lausanne). 2023 Jun 14;14:1163591. doi: 10.3389/fendo.2023.1163591 (PMC10332304; doi:10.3389/fendo.2023.1163591)
Supplement: Supplementary Table 1 — Clinical outcomes of infants comparing GCP vs FCP. [file Table_1.docx]

Supplementary Table 1. Clinical outcomes of infants comparing GCP vs FCP

| Variables | FCP group | GCP group | P value^*^ |
| --- | --- | --- | --- |
|  | (n=40) | (n=11) |  |
| Central line duration (days) | 15.8 ± 6.3 | 12.8 ± 5.1 | 0.150 |
|  | 15 (5-31) | 11 (6-22) |  |
| Length of stay (days) | 24.5 ± 11.9 | 20.7 ± 8.3 | 0.402 |
|  | 23 (8-61) | 20 (12-34) |  |
| Diazoxide initiation (day of life) | 16.6 ± 8.2 | 8 ± 4.2 | 0.004 |
|  | 16.5 (6-32) | 7 (4-17) |  |
| Total intervention days (days) | 32.6 ± 25.1 | 80.2 ± 56.1 | 0.005 |
|  | 21.5 (6-93) | 90 (9-198) |  |
| ^†^Figures reported as mean ± standard deviation and median (minimum – maximum)  ^*^Mann-Whitney U test  FCP, feed-centric pathway; GCP, glucose-centric pathway | | | |
